# Prevalence, knowledge, attitude, motivators and intentional practice of female genital mutilation among women of reproductive age: a community-based analytical cross-sectional study in Tanzania

**DOI:** 10.1186/s12905-023-02356-6

**Published:** 2023-05-04

**Authors:** Charlotte H. Mwanja, Patricia Z. Herman, Walter C. Millanzi

**Affiliations:** School of Nursing and Public Health, Department of Nursing Management and Education, The University of Dodoma, Dodoma, Tanzania

**Keywords:** Female genital mutilation, Women of reproductive age, Knowledge, Attitude, Tanzania

## Abstract

**Background:**

To harmonize and enhance economic growth at the individual, family, community, and national levels, healthy women embody the guardian of family health and a healthy world. They are anticipated to have the freedom to choose their identity in opposition to female genital mutilation in a thoughtful, responsible, and informed manner. Despite restrictive traditions and culture, it is unclear from the available information what exactly would be the drivers of FGM practices in Tanzania from an individual or social perspective. The purpose of this study was to evaluate female genital mutilation among women of reproductive age in terms of its frequency, knowledge, attitudes, and purposeful practice.

**Methods:**

Three hundred twenty-four randomly selected Tanzanian women of reproductive age were studied using a community-based analytical cross-sectional study design quantitatively. Structured questionnaires from earlier studies that were delivered by interviewers were utilized to gather information from the study participants. The statistical software package Statistical Packages for Social Science was used to examine the data. (SPSS v.23). A 5% significance threshold was used with a 95% confidence interval.

**Result:**

A total of 324 women of reproductive age participated in the study with a 100% response rate with a mean age of 25 ± 7.481 years. Findings revealed that 81.8% (*n* = 265) of study participants were mutilated. 85.6% (*n* = 277) of women had inadequate knowledge about FGM, and 75.9% (*n* = 246) had a negative attitude toward it. However, 68.8% (*n* = 223) of them were willing to practice FGM. Their age (36–49 years) (AOR = 2.053; *p* < 0.014; 95%CI: 0.704, 4.325), single women (AOR = 2.443; *p* < 0.029; 95%CI: 1.376, 4.572), never go to school (AOR = 2.042; *p* < 0.011; 95%CI: 1.726, 4.937), housewives (AOR = 1.236; *p* < 0.012; 95%CI: 0.583, 3.826), extended family (AOR = 1.436; *p* < 0.015; 95%CI: 0.762, 3.658), inadequate knowledge (AOR = 2.041; *p* < 0.038; 95%CI: 0.734, 4.358) and negative attitude (AOR = 2.241; *p* < 0.042;95%CI: 1.008, 4.503) were significantly associated to practice female genital mutilation.

**Conclusion:**

The study observed that the rate of female genital mutilation was significantly high and still, women demonstrated the intention to continue practicing it. However, their sociodemographic characteristic profiles, inadequate knowledge, and negative attitude towards FGM were significantly linked with the prevalence. The private agencies, local organizations, the Ministry of Health, and community health workers are alerted to the findings of the current study to design and develop interventions and awareness-raising campaigns for women of reproductive age against female genital mutilation.

**Supplementary Information:**

The online version contains supplementary material available at 10.1186/s12905-023-02356-6.

## Introduction

By 2030, all individuals have a legal right to receive comprehensive sexual and reproductive health information, education, and related healthcare services, such as family planning. This is what Sustainable Development Goal number three (3), Target number 3.7, calls for [1]. The emphasis lies on the health of females and women who represent the cornerstone of the overall health of families, and communities and the improved health of children and adolescents around the globe [2]. Healthy women represent the healthy world and symbolize the custodian of family health that may harmonize and improve the economic growth at the individual, family, community, and national levels respectively [3]. Females and women are expected to have the right to make reasoned, informed, and responsible decisions on the aspect of sexual and reproductive health including when and with whom to be married, and planning when to have how many children at the recommended and healthy birth intervals.

However, studies have shown that oppressive traditions and cultures sometimes deny women and girls access to sexual and reproductive health information, education, and appropriate healthcare services, which can subject them to a variety of health-related burdens, including those that are social-economic, physical, biological, and psychological [4]. Social-related problems such as early marriages, inheritance marriages, and/or female genital mutilation (FGM) just to mention a few have been currently very prominent in developing world Tanzania inclusive than in the developed world. Taking the first position due to its magnitude and health-related burden, female genital mutilation for example is a harmful traditional procedural practice affecting more than 200 million girls and women in 31 countries globally [5]. The term "female genital mutilation" refers to all cultural or scientific practices that include the partial or complete removal of or damage to the external female genitalia for non-medical purposes or to help girls, women, or women's health.

Even though the practice of female genital mutilation is predicted to end globally among girls and women of reproductive age by 2030, it is still prevalent in society, especially in underdeveloped nations [6]. FGM is practiced in 30 African countries, with an estimated three million girls at risk of it each year due to the oppressive traditions/culture against female and women's rights to equal access to sexual and reproductive health information, education, and healthcare services as well as the freedom to make their own decisions about their bodies [7]. Evidence indicates that the victim of FGM procedures may die due to hemorrhage, septic shock, sexual complications, and considerable physical and psychological torture [8]. In addition, females who have undergone genital mutilation may struggle to urinate, develop cysts, contract infections, have an increased risk of losing a baby during delivery, and have a warped sense of who they are. Numerous academics have conducted research in Africa, the Middle East, Southeast Asia, and SSA, as well as in Western nations like Australia, New Zealand, Canada, the United Kingdom, and some parts of Southern America, and they have found that FGM is widely practiced in the countries they have studied, with prevalence rates currently ranging from 1 to 95% [9]. The practice of FGM to mothers and their daughters is most predominantly severe in Eastern Africa including Tanzania, Ethiopia, Djibouti, Eritrea, Somalia, and Sudan which has contributed to negative influences on maternal mortality rates [10]. The factors including geographical location such being living in rural areas are the most commonly identified indicators in the practice of female genital mutilation which is linked with limited educational campaigns to eradicate the practice [11]. Other scholars have shown religion, place of residence, and the occupation of the husband were indicated as the determinants that motivate FGM practice within the community [12]. Intervention, education, information and campaigns, and community participation research have been conducted as the strategies planned in developed countries to eradicate the practice of Female genital mutilation among women and girls [13]. The agencies including WHO, UNICEF, UNFPA, and Global Public Health and Communication agencies have not so far addressed the strategies to eradicate female genital mutilation [14].

The national trend of FGM in Tanzania remains to be a health and social-related problem of concern and it varies across regions of which the highest prevalence of it is found in Manyara (57.7%), Dodoma region (46.7%), Arusha (41%), Mare 32% and Singida region (30.9%) and the existing prevalence in other region are below 15% and those who live in the urban area contributed to (5.3%) of female genital mutilation against those in rural areas [15, 16]. The FGM procedures in the country are mostly practiced in the most common tribes including Gogo, Sandawe, Maassai, Iraq, Chagga, Pare, and Nyaturu tribes that are mostly found in the central and Northern part of Tanzania Mainland [17]. There are several unanswered questions from the literature regarding whether or whether women of reproductive age are properly informed about FGM and its associated negative social, economic, psychological, and/or health-related results while the practice continues in the nation.

The literature has not yet addressed how FGM is perceived by women of childbearing age or its negative effects, the most prevalent type or form of FGM in the nation, or what drives FGM practitioners to carry out their practices despite government and other efforts to stop them. And do sexually active women still intend to practice it or are willing to do so? If this pattern continues, the community will intimidate the human rights of women of reproductive age to sexual and reproductive health by continuing to practice it and raising the costs and hazards of the aforementioned health-related issue. Therefore, the goal of this study is to evaluate the attitudes, knowledge, and purposeful practice of female genital mutilation among Tanzanian women of reproductive age. The baseline data from this study will be used to inform intervention projects or policy changes aimed at preventing FGM in the nation.

## Methods and materials

According to institutional undergraduate and postgraduate norms, this work was completed. I abided by national and international scientific ethics for investigations involving humans or animals.

### Study design and approach

The study was done from March to September 2022 using a community-based analytical cross-sectional study design and a quantitative research methodology. In the rural context of the Dodoma region, the study design was used to measure the prevalence, knowledge, attitude, motivators, and purposeful practice of FGM among women of reproductive age. A single time point was chosen to conduct the study with the happy ladies.

### Study settings

The study was carried out in the Dodoma region, which is home to Tanzania's capital and is situated in the country's center. The region faces an inflow of people through migration because it is the country's newest and fastest-growing metropolis, political, business, and intellectual hub. According to the 2022 census, the region has 3,1687,990 residents, of whom 1.572,865 are women [18]. Even though there are 120 tribes in the Dodoma region, the most well-known indigenous tribes are Gogo, Rangi, and Sandawe. Female genital mutilation among women of reproductive age is one of the second-most prevalent practices in the Dodoma region, making it a purposefully chosen territory. Additionally, with a high incidence of 46.7% female genital mutilation practices in 2021, the region comes in second place in Tanzania. Seven districts make up the area: Chamwino, Chemba, Dodoma Municipal Council, Bahi, Kongwa, Mpwapwa, and Kondoa. To corroborate the claims made in earlier academic works that the majority of FGM procedures are carried out in rural areas, the two districts from rural areas were purposefully sampled for this study.

### Study population

Women who were of reproductive age gave their permission to participate in the study. Other requirements for participation included speaking Swahili well and having lived in the Dodoma region for at least a year. However, women who disclosed being ill or having any medical issue that would hinder conversation were correspondingly disqualified.

### Sample size determination

Based on the recommendations from previous scholars [19, 20], the following procedures were performed to determine the minimum sample size for the study at 95% using the formula by Cochran 1977 [19]. The prevalence used to determine the sample size was obtained from the study conducted by Bargude et al*.*, [21] who observed that 54.9% of wives in families were the key and frontline people who hold a decision power for FGM.

1 $$\mathrm{n}=\frac{{\mathrm{Z}}^{2}\mathrm{p}(1-\mathrm{p})}{{e}^{2}}$$

where;

*n* = sample size,

z = standard normal deviation set at 1.96 (corresponding to a confidence level of 95%),

*p* = prevalence (54.9% ~ 0.549) [21]

e = acceptable marginal error (5% ~ 0.05)

$$\begin{array}{c}\mathrm{n}=\frac{[\left(1.96\right)2\times 0.549\left(1-0.549\right)]}{[\left({0.05}^{2}\right)]}\\ =\frac{[\left(3.8416\right)*0.549(0.451)]}{0.0025}\\ =\frac{0.95117632]}{0.0025}=380\end{array}$$

**Fig. 1:**
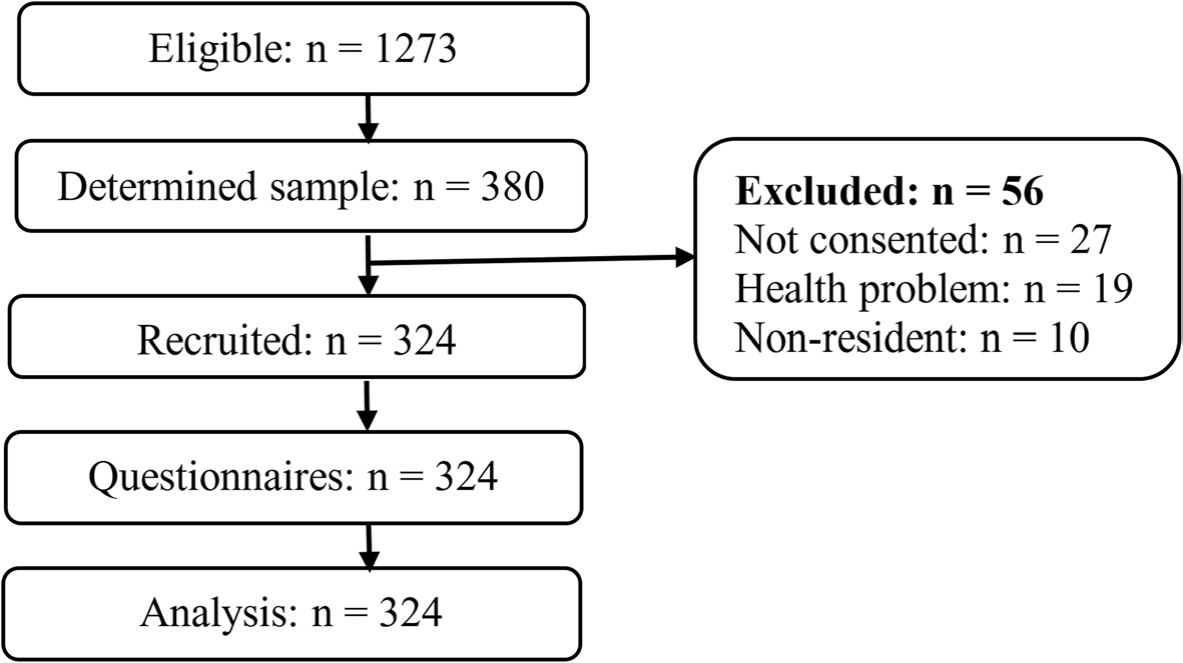
Recruitment procedures of the study respondents. Source: Study plan (2022)

Thus, the minimum sample size of this study was *n* = 380 respondents. However, as indicated in Fig. 1, fifty-six respondents were not involved in the study and therefore, 324 respondents were finally analyzed. As recommended by previous scholars [22, 23], the sample was proportionally distributed in stratum based on the selected secondary schools, classes, and year of study based on the number of students using the proportionate formula [24] $(\mathrm{ni}=\mathrm{Pi}\times \mathrm{n}/\mathrm{P})$.

### Sampling procedures

Due to the increasing prevalence of FGM among teenagers, young adults, and fertile women, the area was purposefully sampled. A total of 324 girls between the ages of 18 and 49 who were of reproductive age were chosen at random using the lottery technique. Based on the wards and villages, the proportional formula was used. Two districts were chosen at random from among the six districts (*n* = 2). There are two wards (*n* = 2) at the district level, and there are two villages in each ward (*n* = 2), for a total of four villages in the study. With an interval of five houses, systematic sampling was employed to identify research participants after the first house was randomly sampled.

### Data collection procedure

Questionnaires were filled out all at once using a face-to-face data-collecting procedure. Data on the prevalence, knowledge, attitude, motivators, and purposeful practice of FGM among women of reproductive age were gathered by the lead investigator with the help of the trained research assistants using standardized questionnaires that were administered by a guided interviewer. To ensure seclusion, unoccupied and separate space that was available in the individual communities was employed. To obtain informed consent and ensure that study participants were willing to participate, the lead investigator briefed study respondents about the study's purpose, benefits, risks, roles, and right to discontinue or continue the study. The respondents were then given brief instructions on how to complete the questionnaire by the primary investigator, and research assistants were on hand to answer any questions, oversee, collect the completed questionnaires, and safeguard them appropriately. To ensure secrecy, respondent names were replaced by codes on the questionnaire. The time required to complete the questionnaires was about between thirty and forty-five minutes.

### Data collection tools and variable measurement

The research data collection methods were adopted and compared to those used in other studies that examined women's knowledge, attitudes, and intentions regarding FGM [10, 25, 26]. The tools were pretested by the lead researcher to ensure they were appropriate for use in Tanzania, and the statistician and experts' colleagues reviewed them for language, clarity, and substance in light of the study population's literacy level. Research materials were then translated into the Swahili language, which was widely spoken in the study sites chosen, to maximize clarity and understanding for the study respondents. The tool consisted of 83 items with four parts including a socio-demographic profile of study participants (*n* = 28 items), knowledge about female genital mutilation (*n* = 21 items), attitude towards female genital mutilation (12 items), and intention to practice female genital mutilation (5 items).

The measurement of variables in this study was informed by previous studies. Knowledge items had “Yes” and “No” responses of which a weight of “1” was assigned to a “Yes” response indicating a correct answer otherwise “0” to the “No” response indicating an incorrect answer. The points were then computed and a cumulative score of 21 points was established of which the highest point was defined as adequate knowledge otherwise, inadequate knowledge. The items for assessing attitudes towards female genital mutilation had a 5-point Likert scale ranging from “1” strongly disagree to “5” points strongly agree. For the descriptive purpose, the attitude items were transformed into interquartile range measurements including “agree”, “neutral” and “disagree” categories. The highest point was considered a “Positive attitude”, the medium point was “Neutral” and the lowest point was considered a “Negative attitude” towards female genital mutilation among women of reproductive age.

The items of intentional practice used to assess FGM were measured using three response items including “Yes”, “No” and “Neutral/I am not sure”. The response of “Yes” was assigned a value of “1” indicating that the women of reproductive age intended to practice FGM. The response of “No” was assigned a value of “0” indicating that the women of reproductive age did not intend to practice FGM. The response of “I am not sure” was assigned a “Neutral response” indicating that the women of reproductive age were not sure if they would practice FGM or not. For the descriptive purpose the intentional practice was transformed into a new variable using interquartile range measurement of which “Yes” was considered as “Willing to practice FGM including”, “No” was considered as “Not willing to practice FGM” and “I am not sure” was considered as “Neutral” means that the women of reproductive age were not sure if they would practice FGM or not.

### Validity and reliability

Contents validity opted for in this study to assure the appropriateness of the research tools which were then shared with a statistician, experts in the field of midwifery/gynecology, and colleagues for their inputs on the aspects of contents appropriateness, relevance, sentence structure, clarity, and organization. Their observations required the research tools to be translated into the Swahili language to blend with the literacy level of the study respondents. A pre-test of the tool was performed by the principal investigator among 30 consented respondents in an independent geographical location from the sampled study settings. Observations from the pilot study indicated that all items were relevant, appropriate, and clear. The questionnaires were filled within the first 30 min during a pilot test in which data were subjected to the scale analysis to determine the reliability measures of the research tools revealed Cronbach alpha = 0.85 for knowledge, 0.90 for attitude, and 0.78 and intentional practice was 0.80. Thus, as recommended by previous scholars [27–29], the tools were reliable for the actual field data collection.

### Data analysis

The Statistical Package for Social Science computer software program version 25 available within the institution was used to analyze data. Socio-demographic characteristics profiles of the study respondents, prevalence, knowledge, attitude, motivators, and intentional practice of female genital mutilation were analyzed descriptively and data were presented in frequencies and proportions. Chi-square and cross-tabulation were used to determine the relationship between variables. As it has been used by previous studies [30, 31] shown in the statistical formula number two (2), a binary and multinomial logistic regression model was performed to determine the association between the predictor variables and the outcome of interest under study at a 95% confidence interval and a 5% significance level.

The following logistic regression model was used

2 $$\left[p=\frac{1}{1+{e-}^{{(b}_{0}+{b}_{1}x)}}\right](\le 0 p\le 1 )$$

whereas; *Ƥ: the* predicted probability of an outcome.

*e:* Exponential

*b*_*0:*_ Constant value

*b*_*1*_*:* Slope

*x:* predictor variable

## Results

### Socio-demographic characteristics of the study participants

The finding in Table 1 revealed that 324 women of reproductive age participated in the study with a 100% response rate. The mean age was 25 ± 7.481 years with 18 to 36 years (75.3%) being the most prominent age group. A 40.7% (*n* = 132) of women were married of which most families were headed by a father (50.6%), while 41.4% (*n* = 134) and 39.8% (*n* = 129) of them had primary education and were housewives (not earning any income). Nevertheless, 49.9% (*n* = 152) of the study respondents engaged themselves in social networks be it religious, traditional, or business groups that would probably be linked with FGM knowledge, attitude, and intentional practices. It was observed that 75.6% (*n* = 245) of them were informed about FGM and 56.5% (*n* = 183) were aware that FGM is being performed in the community and 49.4% (*n* = 160) demonstrated a belief that it is their right to be mutilated.

**Table 1:** The proportional distribution of socio-demographic characteristics among women of reproductive age (*n* = 324)

Items	n(%)
**Age group**	
18–35	244 (75.5)
36–49	80 (34.5)
**Marital status**	
Single	102 (31.5)
Married	132 (40.7)
Cohabiting	69 (21.3)
Divorce	21 (6.5)
**Educational level**	
Primary	134 (41.4)
Secondary	96 (29.6)
Collage and above	36 (11.1)
Never gone to school	58 (17.9)
**Occupation**	
Employed	80 (14.7)
Housewives	129 (39.8)
Self-employed	115 (35.5)
**Types of family**	
Nuclear	126 (38.9)
Extended family	198 (61.1)
**Head of the family**	
Father	164 (50.6)
Mother	96 (29.6)
Father and mother	48 (14.9)
Others	16 (4.9)
**Religion**	
Christian	196 (60.5)
Muslims	128 (39.5)
**Owned a television**	
Yes	144 (44.4)
No	180 (55.6)
**Use of social networking**	
Yes	152 (49.9)
No	172 (53.1)
**Family income**	
Over 1USD	86 (26.5)
Under 1USD	238 (73.5)
**Sources of information**	
**Have you ever heard information about FGM**	
Yes	245 (75.6)
No	79 (24.4)
**Do you have right to be mutilated**	
Yes	160 (49.4)
No	102 (31.5)
Not sure	62 (19.1)
**Do you know people are performing FGM?**	
Yes	183 (56.5)
No	141 (43.5)
**Does your religion allows**	
Yes	164 (50.6)
No	160 (49.4)

Source: Field data (2022)

Key

n: Frequency/count

%: Percentage

≥ 50%: High proportion

### The proportional distribution of the prevalence of female genital mutilation among women of reproductive age in the Dodoma region

**Fig. 2:**
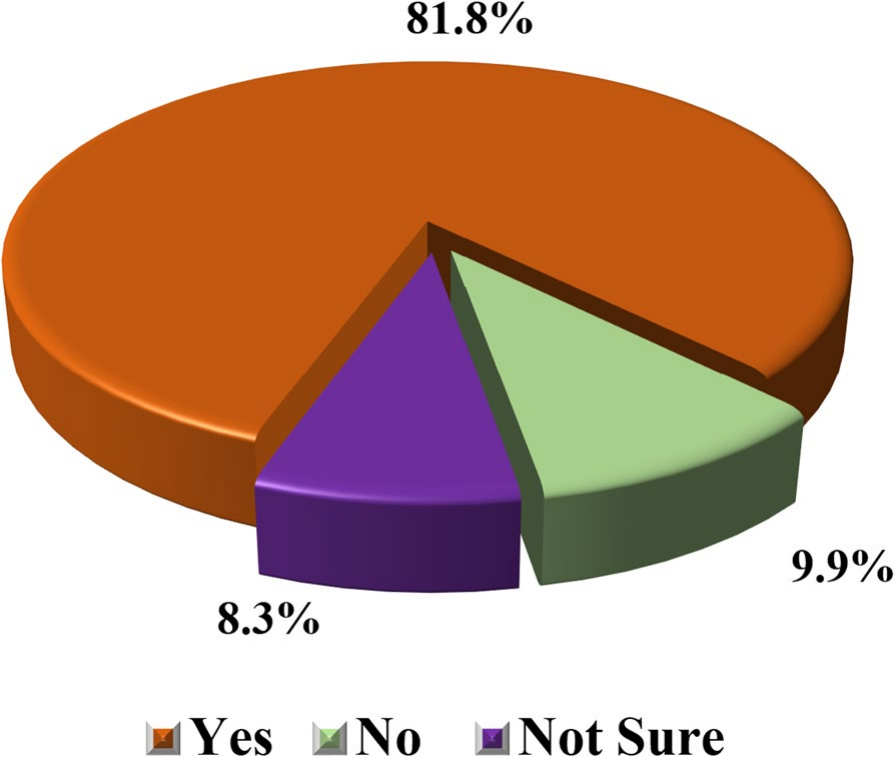
The proportional distribution of prevalence of female genital mutilation among women of reproductive age in Dodoma region (*n* = 324). Source: Field data (2022)

Descriptive analysis was performed to determine the prevalence of female genital mutilation among women of reproductive age. Findings in Fig. 2 show that 81.8% (*n* = 265) of women reported having undergone female genital mutilation, which was significantly high than 9.9% (*n* = 32) and 8.3% (*n* = 27) of those who were not and/or not sure whether they were mutilated or not.

### The proportional distribution of motivators for performing female genital mutilation among women of reproductive age in the Dodoma region

**Fig. 3:**
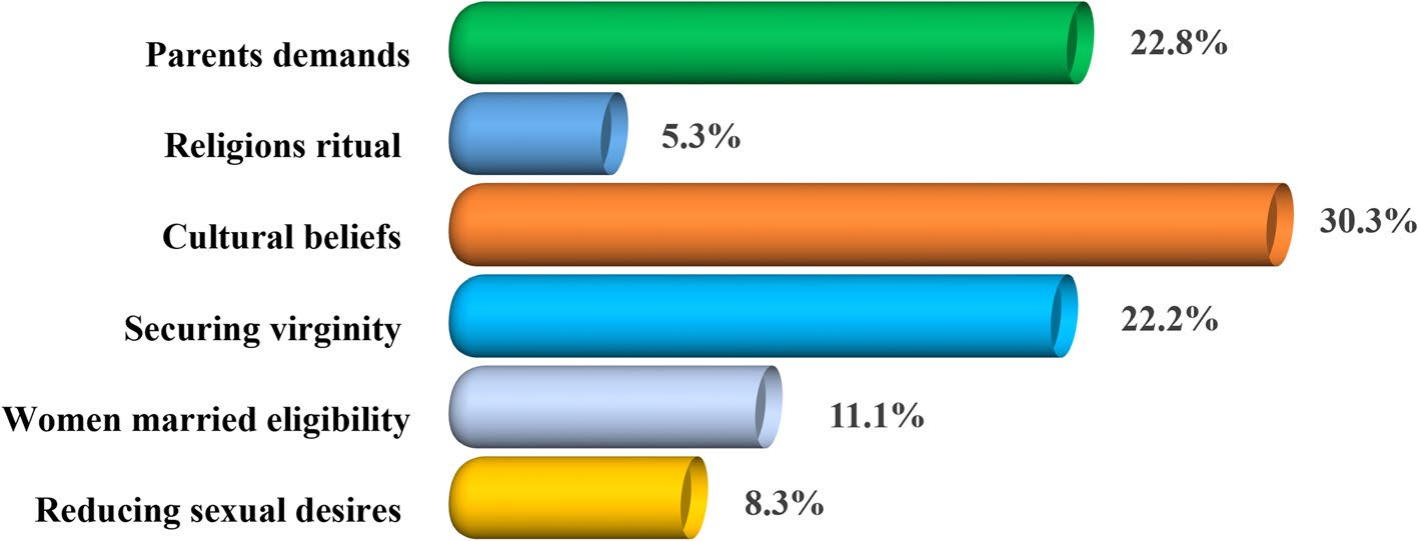
The proportional distribution of the perceived motivators for performing female genital mutilation among women of reproductive age in the Dodoma region (*n* = 324). Source: Field Data (2022)

As shown in Fig. 3, the findings indicated that 30.3% (*n* = 98) of the study respondents reported cultural beliefs as one of the motivators of female genital mutilation while 22.8% (*n* = 74) of them reported parents' demands to be the motivator of female genital mutilation. Moreover, individualized-based characteristics were also assessed and findings revealed that some women of reproductive age reported that securing virginity (22.2%), as a criterion to be married (11.1%), and as a means of reducing sexual desire (8.3%) motivated women of reproductive age into FGM practices. Nevertheless, findings in Fig. 3 indicate that some women reported that women of reproductive age were driven into FGM practices due to religious rituals (5.3%).

### The proportional distribution of the sources of information about female genital mutilation among women of reproductive age in the Dodoma region

**Fig. 4:**
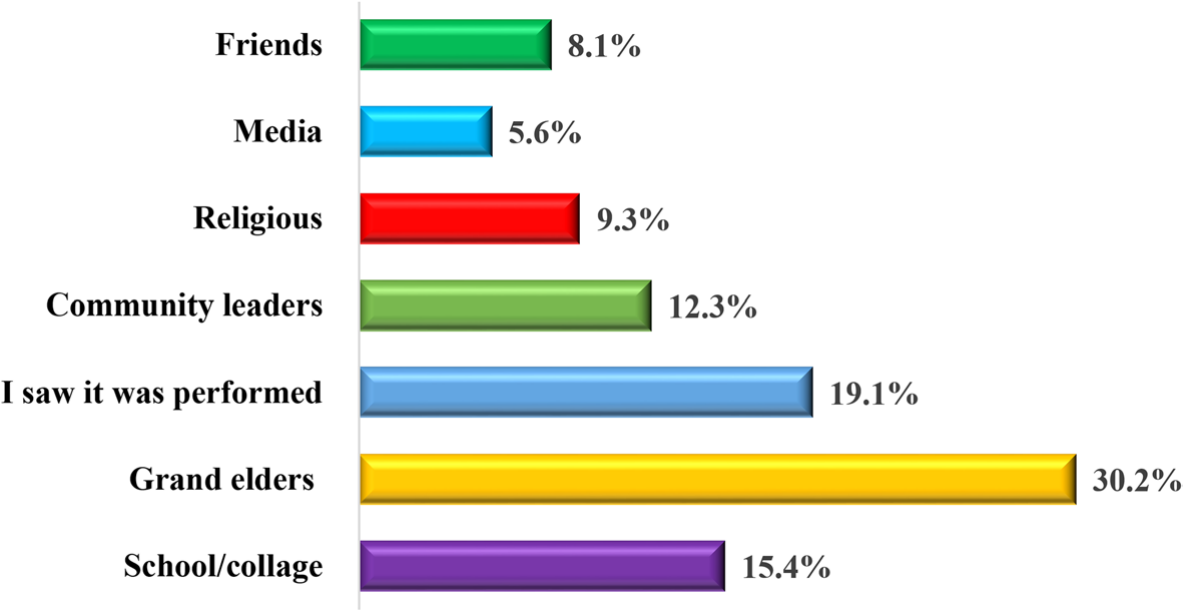
The proportional distribution of the sources of information about female genital mutilation among women of reproductive age in the Dodoma region (*n* = 324). Source: Field Data (2022)

As indicated in Fig. 4, 30.2% (*n* = 98) of the study respondents received information about female genital mutilation from elders while 19.1% (*n* = 62) of them became informed about it when they once observed it being performed on women. Findings yet show that 5.6% (*n* = 18) of the study respondents were informed about FGM from various media, friends (8.1%), religious facilities (9.3%), community leaders (12.3%), and school/college (15.4%).

### The proportional distribution of the reported tools used for female genital mutilation among women of reproductive age in the Dodoma region

**Fig. 5:**
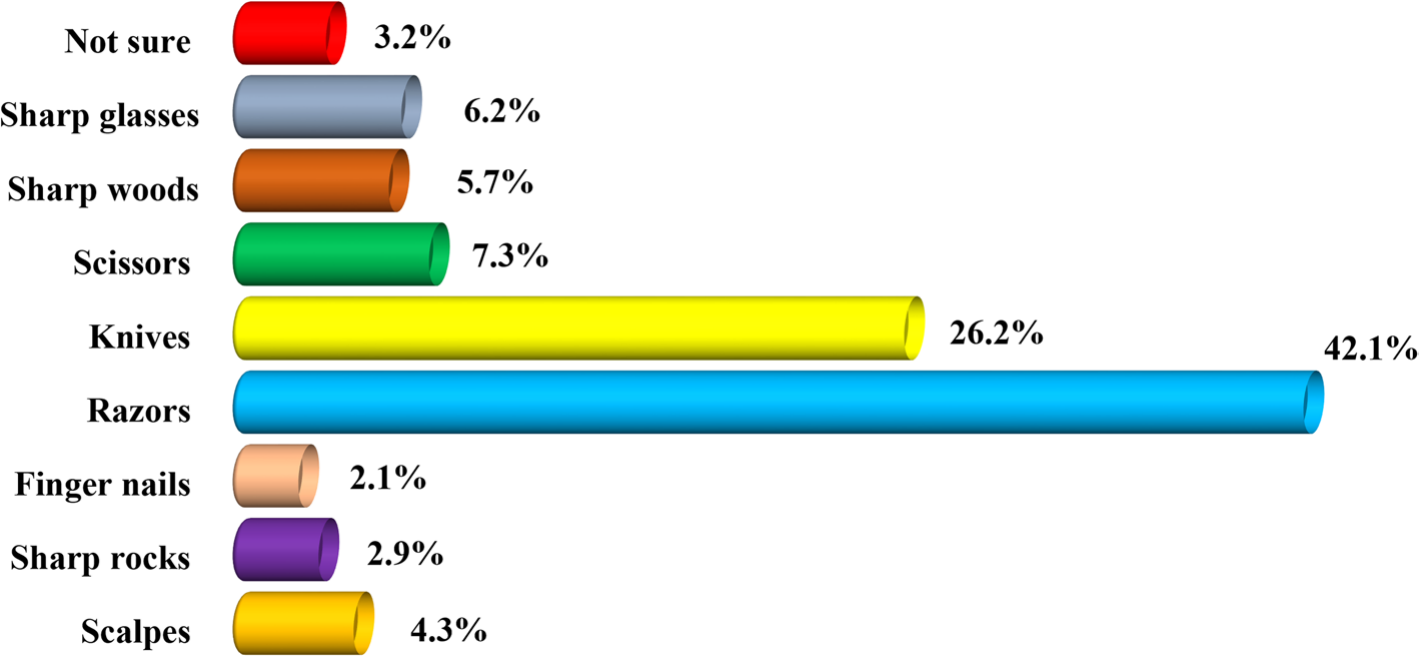
The proportional distribution of the reported tools used to perform female genital mutilation among women of reproductive age in the Dodoma region (*n* = 324). Source: Field Data (2022)

Findings of the reported tools/instruments used to perform the FGM in Fig. 5 demonstrate that 42.1% (*n* = 136) of the study respondents reported razor blades (42.1%), knives (26.5%), scissors (7.3%), sharp glasses (6.2%), sharp woods (7.3%), and scalps (4.3%) as the most commonly used tools/instrument. Other tools included sharp rocks (2.9%) and fingernails (2.1%).

### The proportional distribution of knowledge about female genital mutilation among women of reproductive age in the Dodoma region

**Fig. 6:**
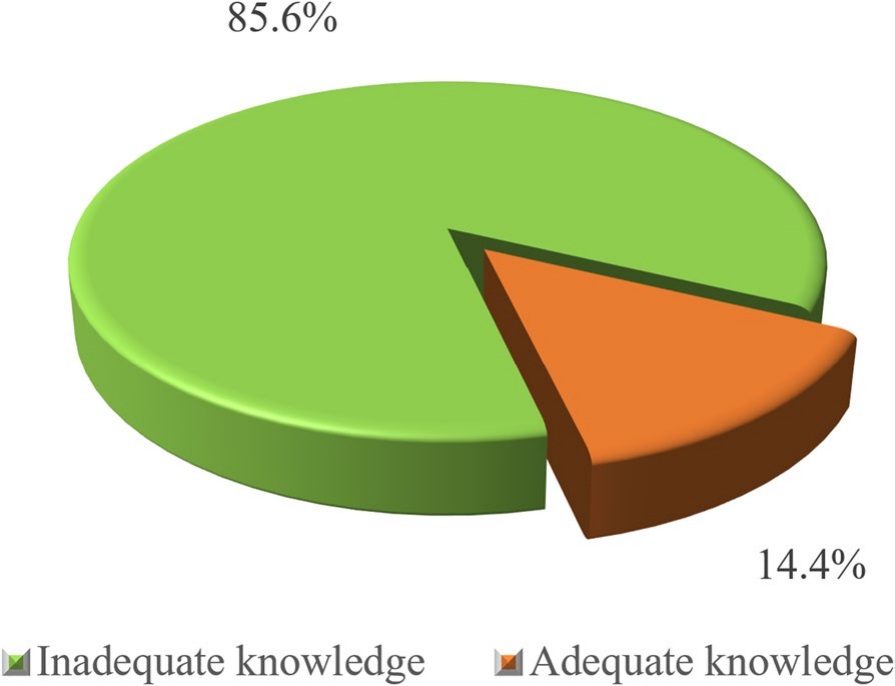
The proportional distribution of the knowledge on female genital mutilation among women of reproductive age in Dodoma region (*n* = 324). Source: Field data (2022)

As shown in Fig. 6, 85.6% (*n* = 277) of the study respondents had inadequate knowledge of female genital mutilation against 14.4% (*n* = 47) who demonstrated adequate knowledge of female genital mutilation.

### The proportional distribution of domains of knowledge about female genital mutilation among women of reproductive age in the Dodoma region

**Fig. 7:**
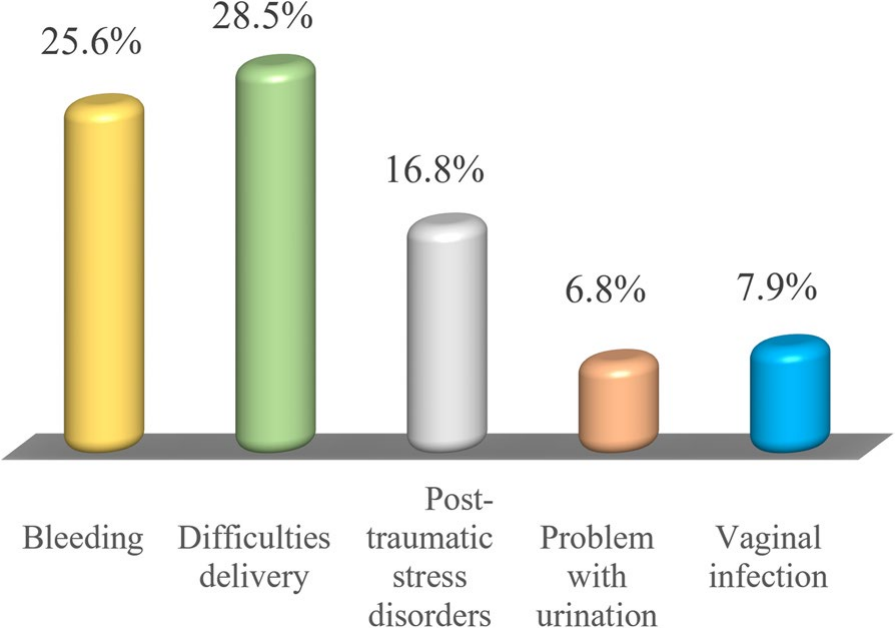
The proportional distribution of knowledge characterization about female genital mutilation among women of reproductive age in Dodoma region (*n* = 324). Source: Field data (2022)

As shown in Fig. 7, 28.5% (*n* = 92) of the study respondents knew that FGM can result in some difficulties during deliveries while 25.6% (*n* = 83) of them knew that FGM can cause severe bleeding. Moreover, 16.8% (*n* = 54) of the respondents knew that being mutilated women of reproductive age would develop posttraumatic stress among them and 7.9% (*n* = 26) of them were knowledgeable that FGM can cause virginal infection while 6.8% (*n* = 22) respondents knew that FGM can lead someone into some urination problems.sult into some difficulties during deliveries.

### The proportional distribution of the attitude on female genital mutilation among women of reproductive age in Dodoma region

**Fig. 8:**
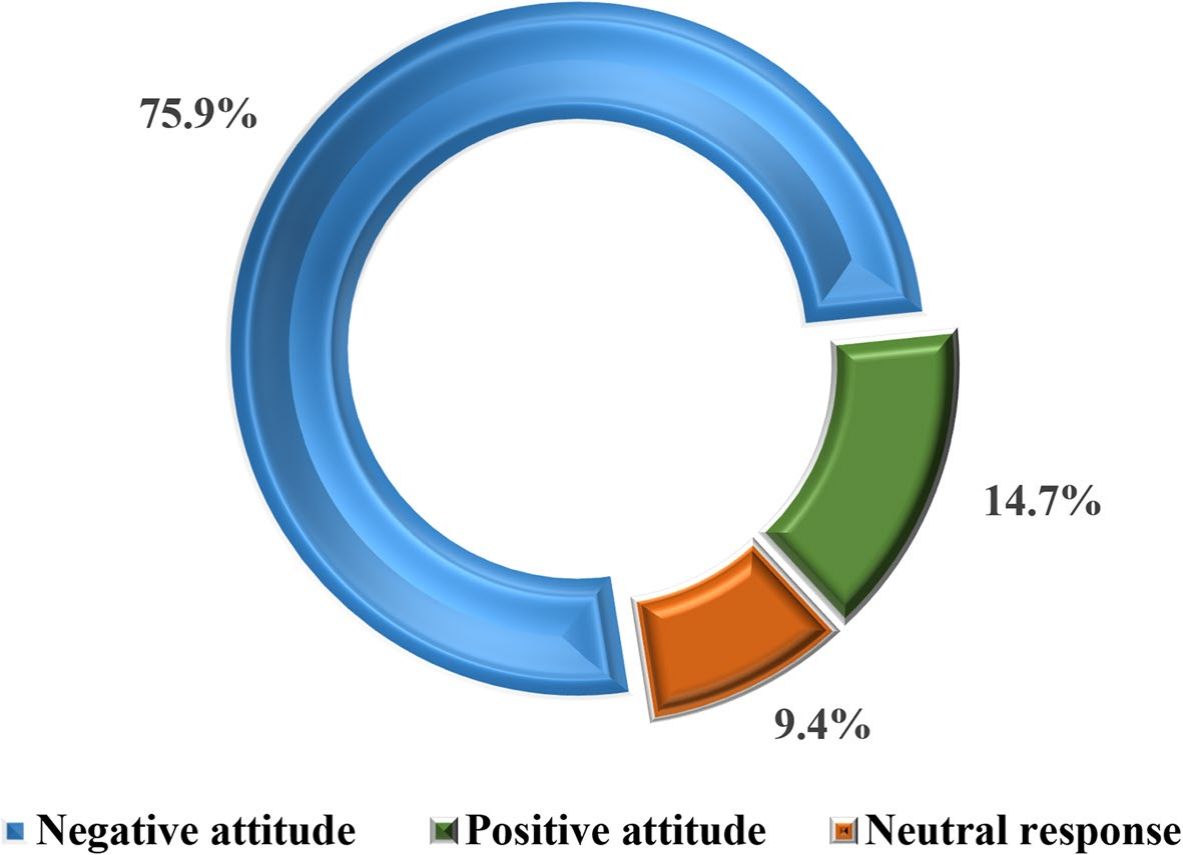
The proportional distribution of attitude about female genital mutilation among women of reproductive age in Dodoma region (*n* = 324). Source: Field Data (2022)

Findings in Fig. 8 show that 75.9% (*n* = 246) of women of reproductive age were found to have a negative attitude towards female genital mutilation against 14.7% (*n* = 48) and 9.4% (*n* = 30) of those who had a positive attitude and were at a neutral point towards FGM practices respectively.

### The proportional distribution of perceived intentional practice of female genital mutilation among women of reproductive

**Fig. 9:**
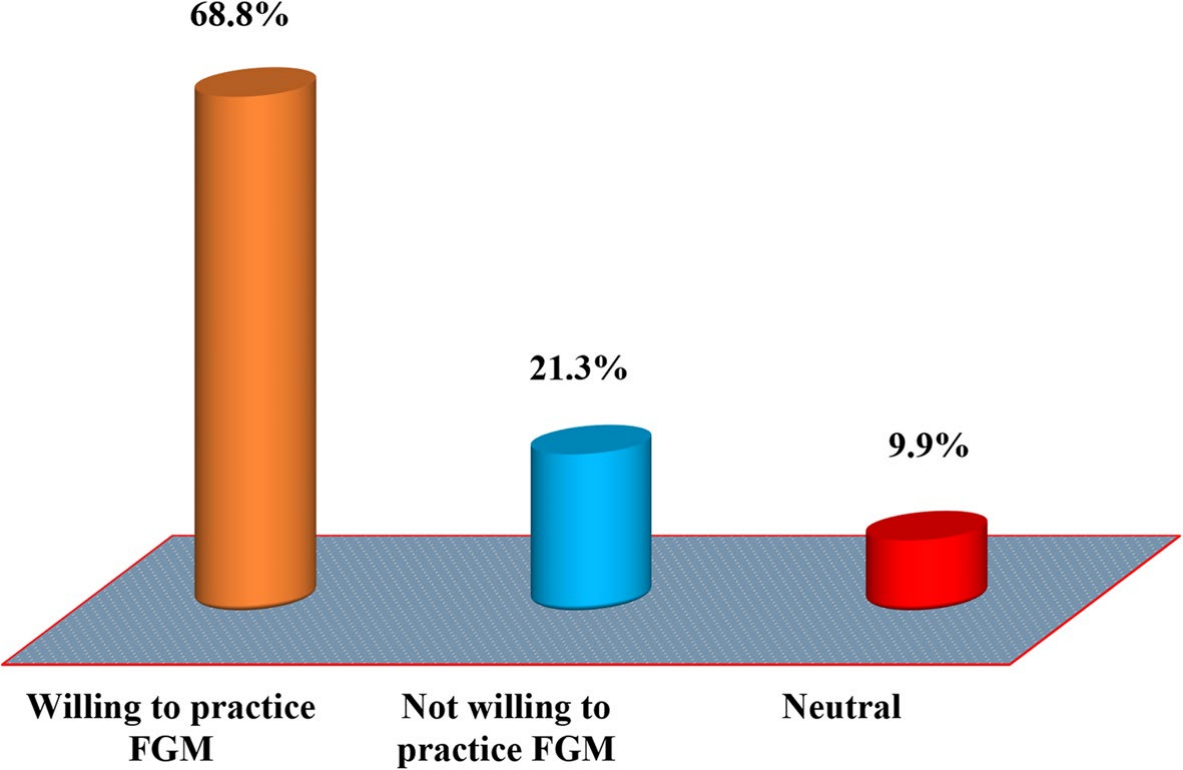
The proportional distribution of perceived intentional practice of female genital mutilation among women of reproductive age in Dodoma region (*n* = 324). Source: Field data (2022)

The study findings in Fig. 9 indicate that 68.8% (*n* = 223) of the study respondents demonstrated a willingness to practice female genital mutilation while 21.3% (*n* = 69) and 9.9% (*n* = 32) of them were not willing to practice it and were at the neutral in deciding to practice female genital mutilation or not respectively.

### The association between the socio-demographic profile characteristic and the level of knowledge on female genital mutilation among women of reproductive age in the Dodoma region

A binary logistic regression analytical model was performed to determine the association between variables at 95%CI and a 5% significance level. As shown in Table 2 some of the socio-demographic characteristics of the study respondents showed to be associated significantly with the level of knowledge regarding FGM among women of reproductive age. The odds of age between 18 -35 years among women of reproductive age was significantly higher over FGM knowledge than another age group of 36–49 years (AOR = 2.815; *p* < 0.032; 95%CI: 0.946, 5.637). Moreover, being married was significantly associated with adequate FGM knowledge among women as compared to those who were single and divorced (AOR = 1.041; *p* < 0.050; 95%CI: 0.303, 3.248). Women with secondary, college, and above education were significantly associated with adequate knowledge about FGM as compared to their counterparts below secondary education (AOR = 2.086; *p* < 0.032; 95%CI: 1.002, 4.165) and (AOR = 1.906; *p* < 0.044; 95%CI: 0.328, 3.342) respectively.

**Table 2:** Factors associated with knowledge about female genital mutilation among women of reproductive age in Dodoma region (*n* = 324)

Variable	COR	*p*-value	95%CI		AOR	*p*-value	95%CI	
			**Lower**	**Upper**			**lower**	**upper**
**Age group**								
18–35 yrs	3.826	0.021	1.196	6.457	2.815	**0.032**	0.946	5.636
36–49 yrs	1							
**Marital status**								
Single	2.755	0.059	1.368	4.526	1.136	0.082	0.850	3.890
Married	3.604	0.047	1.814	5.539	1.041	**0.050**	0.303	3.248
Cohabiting	1							
Divorce	0.840	0.082	0.368	2.897	0.401	0.147	0.030	1.209
**Educational level**								
Primary	2.651	0.071	1.128	4.390	0.129	0.108	0.098	1.025
Secondary	3.890	0.001	1.068	5.890	2.086	**0.032**	1.002	4.165
Collage and above	3.643	0.021	1.393	5.061	1.960	**0.044**	0.328	3.342
Never gone to school	1							
**Occupation**								
Employed	2.564	0.001	1.368	5.856	1.046	**0.031**	0.342	3.184
Housewives	1							
Self-employed	3.654	0.051	1.687	5.174	2.040	0.069	1.076	4.763
**Own TV**								
Yes	2.786	0.001	1.079	4.982	1.798	**0.012**	0.475	3.182
No	1							
**Using social networking**								
Yes	2.864	0.005	1.254	4.795	1.124	**0.023**	0.850	3.036
No	1							
**Head of the family**								
Father	2.351	0.078	1.932	4.456	1.052	0.117	0.683	2.475
Mother	3.259	0.046	1.298	6.604	1.239	0.066	0.746	3.549
Father and mother	2.144	0.024	1.958	4.695	1.063	**0.042**	0.249	3.946
Others	1							
**Religion**								
Christian	2.651	0.024	1.287	5.106	1.547	0.057	0.248	3.294
Muslims	1							
**Family income**								
Over 1USD	2.53	0.015	1.965	4.698	1.032	**0.035**	0.648	3.059
Under 1USD	1							
**Sources of information**								
**Have you ever heard information about FGM?**								
Yes	2.582	0.001	1.387	5.259	1.451	**0.034**	0.681	3.062
No	1							
**Where did you hear FGM for the first time?**								
School/collage	2.265	0.001	1.472	5.047	1.062	**0.042**	0.465	3.841
Health facility	2.676	0.002	1.769	5.083	1.184	**0.043**	0.542	3.853
Adult elders	1							
I saw it performed	2.691	0.060	1.240	4.235	1.443	0.162	0.614	3.986

Source: Field Data (2022)

Key

COR ≥ 1 and *p* < 0.05: positive predictor of the outcome variable when not controlled with other factors

AOR ≥ 1 and *p* < 0.05: positive predictor of the outcome variable when controlled with other factors (The final finding to be reported)

*p* < 0.05: Significant association between variables

Moreover, the odds of being employed were significantly associated with adequate knowledge of FGM practice among women of reproductive age (AOR = 1.046; *p* < 0.031; 95%CI: 0.342, 3.184). Moreover, the odds of the women of reproductive age who owned television and those who are using social media were significantly associated with adequate knowledge of FGM (AOR = 1.798; *p* < 0.012; 95%CI: 0.475, 3.182) and (AOR = 1.124; *p* < value0.024; 95%CI: 0.850, 3.036) respectively. The odds of women of reproductive age who heard information about FGM from the school/college and from the health facility was significantly high over the women’s adequate knowledge of FGM (AOR = 1.062; *p* < 0.042; 95%CI: 0.365, 3.062) and (AOR = 1.184; *p* < 0.043; 95%95%CI: 0.542. 3.853) respectively.

### The association between socio-demographic characteristic profile and attitude on female genital mutilation among women of reproductive health in Dodoma Region

Findings from the multinomial logistic regression analysis show that there was a significant association between some of the socio-demographic characteristics and attitudes of women of reproductive age toward FGM practice. The findings in Table 3 indicate that women aged between 18 and 35 years were more times likely positive attitude towards FGM practices (AOR = 1.052; *p* < 0.023; 95%CI: 0.637, 3.532) against other age groups. Moreover, married women were likely to demonstrate a positive attitude towards FGM practices as compared to their counterparts who were single (AOR = 1.235; *p* < 0.0034; 95%CI: 0.637, 3.349). Likewise, the odds of being educated to college and above and employed were significantly associated with a positive attitude towards FGM practice than other levels of education (AOR = 1.102; *p* < 0.0; 95%CI: 0.748, 3.162) and (AOR = 1.243; *p* < 0.042; 95%CI: 0.649, 3.788) respectively.

**Table 3:** The association between socio-demographic characteristic profile and attitude on female genital mutilation among women of reproductive health in Dodoma Region (*n* = 324)

Variable	COR	*p*-value	95%CI		AOR	*p*-value	95%CI	
			**Lower**	**Upper**			**lower**	**Upper**
**Age group**								
18–35	2.109	0.001	1.715	4.698	1.052	**0.023**	0.654	3.532
36–49	1							
**Marital status**								
Single	2.679	0.055	1.936	4.769	1.384	0.077	0.659	3.965
Married	2.657	0.006	1.615	4.693	1.235	**0.034**	0.637	3.349
Cohabiting	1							
Divorce	2.598	0.056	1.924	4.286	1.225	0.072	0.752	3.971
**Educational level**								
Primary	2.672	0.057	1.279	4.673	1.242	0.063	0.716	3.746
Secondary	3.275	0.051	1.284	5.445	1.939	0.069	0.569	2.849
Collage and above	2.885	0.002	1.582	4.749	1.102	**0.024**	0.748	3.162
Never gone to school	1							
**Occupation**								
Employed	2.452	0.031	1.903	4.042	1.243	0.042	0.649	3.788
Housewives	1							
Self employed	3.092	0.043	1.404	5.941	1.086	0.057	0.595	3.393
**Types of family**								
Nuclear	3.260	0.015	1.437	5.836	1.373	0.031	0.654	3.492
Extended family	1							
**Head of the family**								
Father	1							
Mother	2.983	0.051	1.653	4.572	1.092	0.061	0.737	3.458
Father and mother	2.940	0.016	1.275	4.504	1.048	**0.031**	0.674	3.429
**Own TV**								
Yes	3.084	0.017	1.482	5.142	1.930	**0.034**	0.538	3.048
No	1							
**Using social networking**								
Yes	2.168	0.022	1.053	4.253	1.402	0.043	0.564	3.054
No	1							
**Religion**								
Christian	2.562	0.052	1.476	4.109	1.073	0.061	0.975	3.427
Muslims	1							
**Sources of information**								
**Have you ever heard information about FGM?**								
Yes	2.835	0.022	1.932	5.253	1.092	0.042	0.584	4.070
No	1							

Source: Field Data (2022)

Key

COR ≥ 1 and *p* < 0.05: positive predictor of the outcome variable when not controlled with other factors

AOR ≥ 1 and *p* < 0.05: positive predictor of the outcome variable when controlled with other factors (The final finding to be reported)

*p* < 0.05: Significant association between variables

Findings demonstrate that women from a nuclear type of family and those whose heads of the family were both father and mother were more times likely to have a positive attitude towards FGM practice (AOR = 1.737; *p* < 0.031; 95%CI: 0.654, 3.492) and (AOR = 1.048; *p* < 0.031; 95%CI: 0.674, 3.429) than other family types respectively. The odds of women who owned a television, those who were using social media, and those who had ever heard about FGM were significantly associated with a positive attitude towards FGM practice (AOR = 1.930; *p* < 0.034; 95%CI: 0.538, 3.048), (AOR = 1.902; *p* < 0.043; 95%CI: 0.564, 3.054) and (AOR = 1.092; *p* < 0.042; 95%CI: 0.584, 4.070) than their counterparts respectively.

### The association between socio-demographic characteristic profiles and intentional practice of female genital mutilation among women of reproductive health in Dodoma Region

Table 4 presents the findings from the multinomial regression analytical model that determined the association between variables. It was revealed that women aged between 36 and 49 years were more times likely to demonstrate a willingness to practice FGM than other age groups (AOR = 2.053; *p* < 0.014; 95%CI: 0.704, 4.325). Moreover, findings indicated that the odds of being single (AOR = 2.443; *p* < 0.029; 95%CI: 1.376, 4.572), never gone to school (AOR = 2.042; *p* < 0.011; 95%CI: 1.726, 4.937), housewives (AOR = 1.236; *p* < 0.012; 95%CI: 0.582, 3.826) and extended type of family (AOR = 1.436; *p* < 0.015; 95%CI: 0.762, 3.658) were significantly associated with the intentional practice of FGM among women of reproductive age than other related variables. Besides, women with inadequate knowledge about FGM demonstrated were two times more likely to intend to practice FGM than their counterparts who had adequate knowledge about it (AOR = 2.041; *p* < 0.038; 95%CI: 0.734, 4. 358).

**Table 4:** The association between socio-demographic characteristic profile and intentional practice of female genital mutilation among women of reproductive health in Dodoma Region (*n* = 324)

Variables	COR	*p*-value	95%CI		AOR	*p*-value	95%CI	
			**Lower**	**Upper**			**Lower**	**Upper**
**Age group**								
18–35 yrs	1							
36–49 yrs	2.814	0.005	1.688	5.257	2.053	**0.014**	0.704	4.325
**Marital status**								
Single	3.679	0.015	1.468	5.348	2.443	**0.029**	1.376	4.572
Married	2.975	0.055	1.264	4.174	1.362	0.063	0.573	3.059
Cohabiting	2.549	0.060	1.564	4.164	1.467	0.072	0.783	3.474
Divorce	1							
**Educational level**								
Primary	2.651	0.053	1.255	4.492	1.742	0.069	0.605	3.843
Secondary	2.629	0.056	1.263	4.258	1.362	0.060	0.864	3.436
Collage and above	1							
Never gone to school	3.632	0.002	2.423	5.144	2.042	**0.011**	1.726	4.937
**Occupation**								
Employed	1							
Housewives	2.596	0.006	1.654	4.139	1.236	**0.012**	0.582	3.826
Self employed	2.765	0.031	1.596	4.748	1.031	0.057	0.761	3.674
**Types of family**								
Nuclear	1							
Extended family	2.283	0.002	1.453	4.562	1.436	**0.015**	0.762	3.658
**Head of the family**								
Father	1							
Mother	3.216	0.009	1.253	5.435	2.148	**0.021**	1.641	4.146
Father and mother	2.854	0.058	1.136	4.586	1.264	0.073	0.673	3.572
**Knowledge**								
Adequate	1							
Inadequate	3.258	0.021	1.286	6.261	2.041	**0.038**	0.734	4.358
**Attitude**								
Positive	1							
Negative	3.546	0.023	1.041	5.476	2.241	**0.042**	1.008	4.503

Source: Field Data (2022)

Key

COR ≥ 1 and *p* < 0.05: positive predictor of the outcome variable when not controlled with other factors

AOR ≥ 1 and *p* < 0.05: positive predictor of the outcome variable when controlled with other factors (The final finding to be reported)

*p* < 0.05: Significant association between variables

Nevertheless, women with a negative attitude towards FGM practice had higher odds (AOR = 2.241; *p* < 0.042; 95%CI: 1.008, 4.503) to intend practicing it as compared to those who had a positive attitude towards it.

## Discussion

The study discovered that the prevalence of female genital mutilation was noticeably high among the group under investigation. Even though many women of reproductive age had heard about FGM and its negative effects on health via friends, the media, religious institutions, elders, and school, and having seen a friend, neighbor, or even oneself being mutilated, the prevalence of the practice was still alarmingly high. The most often used and reported tools/equipment included sharp objects (glasses, wood, rocks), scissors, blades, scalpels, and fingernails, which may not be safe enough for FGM in healthy women. Many women were aware that FGM is strongly associated with some bad health outcomes, such as excessive bleeding, difficulties giving birth, post-traumatic stress disorders, urinary issues, and/or vaginitis. However, the vast majority of women expressed intentions to keep doing it.

Parents' demands for FGM, religious practices, cultural values, and personal ideologies that use it to secure virginity, suppress sexual urges, or meet marriage requirements all appear to exist and will likely continue to put women at risk for the aforementioned health-related outcomes. However, this study found that poor knowledge, hostility toward FGM, being between the ages of 36 and 50, living alone, not having attended school, being a housewife, living in extended families, and living in families headed by mothers were all associated with increased likelihood of engaging in FGM.

Similar findings to this study were observed in other previous studies such as research that was conducted by Bargude et al*.*, [21] in Ethiopia, which uncovered that the highest proportion of the studied women of reproductive age (88.9%) were mutilated. Age and hyperactivity were significantly linked with the prevalence of FGM among them. They argued that administrative organs and community-based interventions would be given priority and implemented effectively to discourage risky drivers including traditions. Moreover, a scholarly work by Mohammed et al*.*, [25] in Agarf town revealed that the majority (86.1%) of the women of reproductive age were undergone FGM. Their study found a strong correlation between the frequency of FGM and women's knowledge, attitudes, religious beliefs, cultural practices, and/or oppressive customs.

They said that strategic innovations needed to be given top priority to arm the community with knowledge and a positive outlook so they may decide against FGM in a reasoned, responsible, and informed manner. Other scholars (Tammary et al*.*, [32]) conducted a systematic narrative synthesis in Africa on mental and sexual health outcomes associated with FGM. Their research revealed that a sizable portion of the reproductively healthy women who were mutilated went through more stressful times in life than those who weren't. Age, education, knowledge, and/or attitude are some factors that increase the severity of the issue, and mutilated women were more likely to experience psychiatric episodes of despair, anxiety, post-traumatic stress disorder, and sleep difficulties.

The findings observed by Omigbodun et al*.*, [8] on the women’s psychological perspectives and experiences of the FGM in Southeast Nigeria indicated that FGM was considered a traditional issue that was mandatory, and thus, if a woman was not mutilated she felt shame, stigmatized and most of them listed FGM as of no adverse health-related outcomes to their daily living. They recommended higher authorities collaborate with communities on re-thinking innovative and sustainable strategies and interventions against FGM. Moreover, similar findings were reported by O’Neill et al*.*, [33] which indicated that psychological and physiological impacts associated with female genital mutilation included poor self-image, failure to thrive, and an increased tendency toward the development of depression with the description that the survivors of FGM struggled both physically and psychologically.

Findings of this study also, tally with those of a school-based interventional study done in Sudan by Mahgoup et al*.*, [22] which indicated that there was a significant impact between when a woman is empowered with knowledge and positive attitude against FGM practices or intention to practice it later in their lives. This suggests that to reverse the trend of FGM, interventions and other strategic plans should concentrate on and promote the rights of women and the general public to sexual and reproductive health information, education, and related healthcare services. As most of the reviewed studies were conducted in developing nations that blend well with Tanzania in terms of cultural issues and other similar social demographic characteristics profiles, similarities between the study at hand and some prior scholarly works may be attributable to the same context, study population, and geographic location.

According to the observed results, it may be necessary to teach young girls in their primary and secondary education levels so that they will be equipped with the knowledge and a good attitude toward FGM. Homes, hospitals, schools, healthcare institutions, and places of worship play a significant role in educating women of reproductive age about FGM and its related psychological and physical repercussions.

## Conclusion

In this study, it was discovered that women of reproductive age had a greater rate of female genital mutilation. However, it was discovered that the majority of the women lacked adequate knowledge, particularly regarding the complications of FGM, had unfavorable attitudes toward the procedure, and yet showed signs of wanting to engage in FGM. Age, marital status, educational attainment, social media and networking use, family type, the health of the family, information sources, and family income all seemed to have a substantial impact on the variables under consideration. To end the growing and pervasive trend of FGM among women of reproductive age, health policies, health-related promotion activities, and campaigns may need to concentrate on people's sociodemographic characteristics profiles and the society at large.

## Recommendation

Governments, health facilities, NGOs, and community health workers have to initiate continuum and sustainable legal measures against female genital mutilation. The women are suffered physically and psychologically with the unrepaired and unrecovered long-term complications that are associated with the practice of female genital mutilation. The government through the Ministry of Health should advocate health strategic plans on the aspect of controlling and harmonizing health education provided by traditions, media, schools, health facilities, and/or religious facilities against FGM. The current study also recommends the development of interventional studies to empower women of reproductive age with knowledge and a positive attitude toward the fight against FGM.

## Supplementary Information

**Additional file 1.** English version questionnaires.

## Data Availability

Data will be available under special request at patriciaz1006@gmail.com.

## Abbreviations

FGM: Female genital mutilation
UNICEF: United Nations Children’s Fund
WHO: World Health Organization
SDGs: Sustainable Development Goals
UNFPA: United Nations of Population Funds
TV: Television
USD: United State Dollar
