# Supplementary material for: Prevalence, knowledge, attitude, motivators and intentional practice of female genital mutilation among women of reproductive age: a community-based analytical cross-sectional study in Tanzania
Source: BMC Womens Health. 2023 May 4;23:226. doi: 10.1186/s12905-023-02356-6 (PMC10158332; doi:10.1186/s12905-023-02356-6)
Supplement: Supplementary file 1 — Additional file 1. English version questionnaires. [file 12905_2023_2356_MOESM1_ESM.docx]

**THE UNIVERSITY OF DODOM**


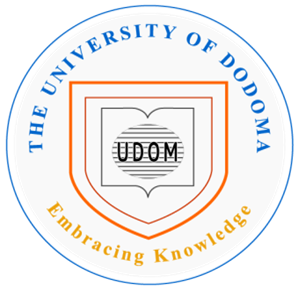


**School of Nursing and Public Health**

**Department of Clinical Nursing**

**An Interviewer administered Questionnaires about Prevalence, knowledge, attitude, motivators and intentional practice of female genital mutilation among women of reproductive age: A community-based analytical cross-sectional study in Tanzania**

**Instructions**

1. Read carefully before answering the questions.
2. Do not write your name in the paper provided.
3. Put tick (v) in the space provided.
4. Please return up your paper to the responsible person on completion.

**English Version Questionnaires**

**SECTION A:** **Socio-demographic Characteristics of the Study Participants**

The following questions under this part are categorized into personal information including yourself, family matters, religiosity, social matters, economical issues, and academic issues. Please read and circle the answer that corresponds to you.

**Items**

1. Age in years …………………..
2. Marital status
3. Single ( )
4. Married ( )
5. Cohabiting ( )
6. Divorce ( )
7. Educational level
8. Primary ( )
9. Secondary ( )
10. Collage and above ( )
11. Never gone to school ( )
12. Occupation of the study participants
13. Employed ( )
14. Housewives ( )
15. Self-employed ( )
16. Types of family
17. Nuclear
18. Extended family
19. Head of the family
20. Father ( )
21. Mother ( )
22. Father and mother ( )
23. Others ( )
24. Religion
25. Christian ( )
26. Muslims ( )
27. Do you own Television?
28. Yes ( )
29. No ( )
30. Use of social networking
31. Yes ( )
32. No ( )
33. Family income
34. Over 1USD ( )
35. Under 1USD ( )
36. Have you ever heard information about Female genital mutilation?
37. Yes ( )
38. No ( )
39. I am not sure ( )
40. Do you have the right to be mutilated?
41. Yes
42. No
43. I am not sure
44. Do you know people who are performing Female genital mutilation?
45. Yes ( )
46. No ( )
47. I do not know ( )
48. Does your religion allows the practice of female genital mutilation?
49. Yes ( )
50. No ( )
51. I do not know ( )
52. Have you ever been mutilated?
53. Yes ( )
54. No ( )
55. Not sure ( )

**SECTION B: Perceived motivators to female genital mutilation**

What do you think are the motivators that contribute to female genital mutilation?

1. Poverty
2. Yes ( )
3. No ( )
4. I am not sure ( )
5. Individual interest/preferences
6. Yes ( )
7. No ( )
8. I am not sure ( )
9. The government demand
10. Yes ( )
11. No ( )
12. I am not sure ( )
13. Parents demand
14. Yes ( )
15. No ( )
16. I am not sure ( )
17. Religious rituals
18. Yes ( )
19. No ( )
20. I am not sure ( )
21. Cultural beliefs
22. Yes ( )
23. No ( )
24. I am not sure ( )
25. Social media
26. Yes ( )
27. No ( )
28. I am not sure ( )
29. Securing virginity
30. Yes ( )
31. No ( )
32. I am not sure ( )
33. Prevention of sexually transmitted infections
34. Yes ( )
35. No ( )
36. I am not sure ( )
37. Women married eligibility
38. Yes ( )
39. No ( )
40. I am not sure ( )
41. Peer pressure
42. Yes ( )
43. No ( )
44. I am not sure ( )
45. Reducing sexual desires
46. Yes ( )
47. No ( )
48. I am not sure ( )
49. Others (Specify) ………………………………………………………………….

**SECTION C: Sources of information**

**Where did you first hear the pieces of information about female genital mutilation?**

1. Biological parents
2. Yes ( )
3. No ( )
4. Friends
5. Yes ( )
6. No ( )
7. Media
8. Yes ( )
9. No ( )
10. Religious
11. Yes ( )
12. No ( )
13. Community leaders
14. Yes ( )
15. No ( )
16. I saw it was performed
17. Yes ( )
18. No ( )
19. Grand elders
20. Yes ( )
21. No ( )
22. Schools/collage
23. Yes ( )
24. No ( )
25. Others (Specify) ………………………………………………………………….

**SECTION D: Resources used to perform female genital mutilation**

What equipment do you think is used for female genital mutilation?

1. Sharp glasses
2. Yes ( )
3. No ( )
4. Not sure ( )
5. Sharp woods
6. Yes ( )
7. No ( )
8. Not sure ( )
9. Scissors
10. Yes ( )
11. No ( )
12. Not sure ( )
13. Knives
14. Yes ( )
15. No ( )
16. Not sure ( )
17. Razors blade
18. Yes ( )
19. No ( )
20. Not sure ( )
21. Fingernails
22. Yes ( )
23. No ( )
24. Not sure ( )
25. Sharp rocks
26. Yes ( )
27. No ( )
28. Not sure ( )
29. Others (Specify) ………………………………………………………………….

**SECTION E:** **Question on Knowledge**

1. Do you know anything about female genital mutilation?
2. Yes ( )
3. No ( )
4. I do not know ( )
5. What if female genital mutilation?
6. …………………. ( )
7. …………………. ( )
8. ………………… ( )
9. …………………. ( )
10. Does female genital mutilation among females and women equivalent to circumcision to boys?
11. Yes ( )
12. No ( )
13. I do not know ( )
14. Is it recommended for all female and women to be genitally mutilated?
15. Yes ( )
16. No ( )
17. I do not know ( )
18. Do you know that female genital mutilation is against human rights
19. Yes ( )
20. No ( )
21. I do not know ( )
22. Do you know that female and women have the right to say “No” to female genital mutilation?
23. Yes ( )
24. No ( )
25. I do not know ( )
26. Do you know a female genital can lead someone into health-related effects?
27. Yes ( )
28. No ( )
29. I do not know ( )
30. Is pain is one of the effects of female genital mutilation?
31. Yes ( )
32. No ( )
33. I do not know ( )
34. Is the Culture and pride are the possible causes of female circumcision?
35. Yes ( )
36. No ( )
37. I do not know ( )
38. Does poverty fuels female genital mutilation practices?
39. Yes ( )
40. No ( )
41. I do not know ( )
42. Is it true that girls of reproductive age about 15-20 years are more likely to suffer with female genital mutilation?
43. Yes ( )
44. No ( )
45. I do not know ( )
46. Is it right during harvest most people should practice female genital mutilation
47. Yes ( )
48. No ( )
49. I do not know ( )
50. Is it true that girls are more likely to suffer with FGM than women?
51. Yes ( )
52. No ( )
53. I do not know ( )
54. Performing female circumcision brings pride
55. Yes ( )
56. No ( )
57. I do not know ( )
58. Urinating problems among female and women can be contributed by female genital mutilation?
59. Yes ( )
60. No ( )
61. I do not know ( )
62. Can female genital mutilation cause sexually transmitted infections (STIs) including Human immunodeficiency virus (HIV)?
63. Yes ( )
64. No ( )
65. I do not know ( )
66. Can female genital mutilation distort personal identity?
67. Yes ( )
68. No ( )
69. I do not know ( )
70. Can female genital mutilation cause urinary tract infections (UTI)?
71. Yes ( )
72. No ( )
73. I do not know ( )
74. Can female genital mutilation cause cysts later in life?
75. Yes ( )
76. No ( )
77. I do not know ( )
78. Maternal and/or newborn deaths is one of the negative outcomes of performing female circumcision
79. Yes ( )
80. No ( )
81. I do not know ( )
82. Can female genital mutilation practices be eliminated in the society?
83. Yes ( )
84. No ( )
85. I do not know ( )

**SECTION F: Questions on Attitudes of Individuals**

1. The government and other stakeholders should promote female genital mutilation practices
2. Agree ( )
3. Strongly agree ( )
4. Disagree ( )
5. Strongly disagree ( )
6. Neutral ( )
7. Female genital mutilation improves personal identity in the community/society
8. Agree ( )
9. Strongly agree ( )
10. Disagree ( )
11. Strongly disagree ( )
12. Neutral ( )
13. Families should continue to practice female genital mutilation
14. Agree ( )
15. Strongly agree ( )
16. Disagree ( )
17. Strongly disagree ( )
18. Neutral ( )
19. If you become pregnant immediately and not circumcised, you have high risk during delivered
20. Agree ( )
21. Strongly agree ( )
22. Disagree ( )
23. Strongly disagree ( )
24. Neutral ( )
25. Female genital mutilation increases beautifulness among girls
26. Agree ( )
27. Strongly agree ( )
28. Disagree ( )
29. Strongly disagree ( )
30. Neutral ( )
31. Female genital mutilation helps to stop curse in the family
32. Agree ( )
33. Strongly agree ( )
34. Disagree ( )
35. Strongly disagree ( )
36. Neutral ( )
37. Women who undergo circumcision and gets pregnancy are considered strong
38. Agree ( )
39. Strongly agree ( )
40. Disagree ( )
41. Strongly disagree ( )
42. Neutral ( )
43. I feel incomplete women if I did not undergo female circumcision
44. Agree ( )
45. Strongly agree ( )
46. Disagree ( )
47. Strongly disagree ( )
48. Neutral ( )
49. Female circumcision influence married women to stay in their marriage.
50. Agree ( )
51. Strongly agree ( )
52. Disagree ( )
53. Strongly disagree ( )
54. Neutral ( )
55. Female genital mutilation increases desire to sexual intercourses
56. Agree ( )
57. Strongly agree ( )
58. Disagree ( )
59. Strongly disagree ( )
60. Neutral ( )
61. A mutilated girl have equal and increased chance to be married
62. Agree ( )
63. Strongly agree ( )
64. Disagree ( )
65. Strongly disagree ( )
66. Neutral ( )
67. Your mother or husband must instruct you to initiate female genital mutilation
68. Agree ( )
69. Strongly agree ( )
70. Disagree ( )
71. Strongly disagree ( )
72. Neutral ( )

**SECTION G: Questions on Intentions to Practice Female Circumcision**

1. Do you think your girl children will undergo female genital mutilation when they reach the recommended time?
2. Yes ( )
3. No ( )
4. I am not sure ( )
5. Do you and/or your young girls plan to be mutilated to get married easily?
6. Yes ( )
7. No ( )
8. I am not sure ( )
9. Do you plan to advise your neighbors and friend to be mutilated?
10. Yes ( )
11. No ( )
12. I am not sure ( )
13. Do you think you can say “No” to female genital mutilation and stick to it?
14. Yes ( )
15. No ( )
16. I am not sure ( )
17. If someone forces you or your daughter to be mutilated. What will you be able to say “No” and stick to it?
18. Yes ( )
19. No ( )
20. I am not sure ( )
